# Cross-network interaction for diagnosis of major depressive disorder based on resting state functional connectivity

**DOI:** 10.1007/s11682-020-00326-2

**Published:** 2020-07-30

**Authors:** Xueling Zhu, Fulai Yuan, Gaofeng Zhou, Jilin Nie, Dongcui Wang, Ping Hu, Lirong Ouyang, Lingyu Kong, Weihua Liao

**Affiliations:** 1 Department of Radiology, Xiangya Hospital, Central South University, Changsha, China; 2 National Clinical Research Center for Geriatric Disorders, Xiangya Hospital, Central South University, Changsha, China; 3 Health Management Center, Xiangya Hospital, Central South University, Changsha, China

**Keywords:** Major depressive disorder, Multivariate pattern analysis, Resting-state fMRI, Functional connectivity, Cross-network interaction

## Abstract

Previous studies have suggested that resting-state functional connectivity plays a central role in the physiopathology of major depressive disorder (MDD). However, the individualized diagnosis of MDD based on resting-state functional connectivity is still unclear, especially in first episode drug-naive patients with MDD. Resting state functional magnetic resonance imaging was enrolled from 30 first episode drug-naive patients with MDD and age- and gender-matched 31 healthy controls. Whole brain functional connectivity was computed and viewed as classification features. Multivariate pattern analysis (MVPA) was performed to discriminate patients with MDD from controls. The experimental results exhibited a correct classification rate of 82.25% (*p* < 0.001) with sensitivity of 83.87% and specificity of 80.64%. Almost all of the consensus connections (125/128) were cross-network interaction among default mode network (DMN), salience network (SN), central executive network (CEN), visual cortex network (VN), Cerebellum and Other. Moreover, the supramarginal gyrus exhibited high discriminative power in classification. Our findings suggested cross-network interaction can be used as an effective biomarker for MDD clinical diagnosis, which may reveal the potential pathological mechanism for major depression. The current study further confirmed reliable application of MVPA in discriminating MDD patients from healthy controls.

## Introduction

As one of the most common psychiatric disorders worldwide, major depressive disorder (MDD) is characterized by persistent, pervasive feelings of sadness, guilt, and worthlessness, which leads to serious economic impact to the families and bring great burden to the society (World Health Organization 2017). However, the diagnosis of MDD is still challenging because the diagnosis is primarily based on both the patient’s self-reported symptoms and the psychiatrist’s experience (American Psychiatric Association 2013). Reliable MDD detection becomes difficult especially in the condition without experienced psychiatrist, which limits subsequent treatment of this disease (Mitchell et al. 2009; Nabbe et al. 2017). Obviously, it is necessary to develop an automated and objective method to help to diagnosis MDD.

In the last decade, multi-modal magnetic resonance imaging (MRI) techniques have been widely used to characterize the underlying pathophysiology of mental diseases (Buckner 2010). Compared with the task MRI, resting-state fMRI has attracted considerable attention owing to easier implementation and fewer requirements to the patients (Greicius et al. 2003; Fox and Raichle 2007). Although some studies have focused on investigating dynamic functional connectivity (Li et al. 2019; Liao et al. 2019) or combined dynamic and static connectivity (Liao et al. 2018), resting-state functional connectivity (static) has always been proven effective in revealing the alterations of brain functional networks in neuropsychiatric disorders including Alzheimer (Badhwar et al. 2017), schizophrenia (Dong et al. 2018) and depression (Greicius et al. 2007; Greicius 2008).

The default mode network (DMN) refers to some brain areas that form an integrated system for self-related activity, including autobiographical, self-monitoring and social functions, which mainly contained the medial prefrontal cortex, anterior cingulate cortex, posterior cingulate cortex, inferior parietal lobule, parahippocampal gyrus and hippocampus (Raichle et al. 2001). The salience network (SN) usually is involved in processing emotion or monitoring for salient events (Seeley et al. 2007), which included insula, amygdala, temporal poles, super temporal gyrus, pallidum and caudate (Menon 2011). The central executive network (CEN) is responsible for high-level cognitive functions, notably the control of attention and working memory (Mulders et al. 2015), which were anchored in dorsolateral prefrontal cortex, and posterior parietal cortex (Seeley et al. 2007; Habas et al. 2009). Although findings are somewhat inconsistent, previous studies have revealed that the pathophysiology of MDD involves a large-scale dysfunction in brain functional networks such as DMN, SN and CEN (Greicius et al. 2007; Sexton et al. 2012; Zhu et al. 2012; Hamilton et al. 2013; Guo et al. 2014b; Manoliu et al. 2014a). However, most of these studies traditionally adopt the univariate analysis, which has neglect the highly interconnected nature of the brain (Davatzikos, 2004). Whether altered resting-state functional connectivity could be used in the individualized diagnosis of MDD is still unknown.

How to differentiate MDD at the individual level is the key problem to be settled. With the incoming of artificial intelligence era, machine learning methods have been used widely in brain image analysis (Liu et al. 2012; Smith 2012; Liu et al. 2015).As one of the typical supervised machine learning methods, multivariate pattern analysis (MVPA) can extract stable identification features from brain image data to differentiate patients from healthy controls at the individual subject level (Orru et al. 2012; Wolfers et al. 2015). In contrast to the univariate statistical methods, MVPA could further detect exciting spatially distributed information to highlight neural mechanisms of psychiatric disease. An increasing number of neuroimaging studies focused on applying MVPA to discriminate MDD patients from healthy controls (Fu et al. 2008; Craddock et al. 2009; Liu et al. 2012; Zeng et al. 2012; Ma et al. 2013; Zhong et al. 2017). A recent meta-analysis of multivariate pattern recognition studies to differentiate patients diagnosed with MDD from healthy controls has confirmed high representational capacity of MVPA methods to identify neuroimaging-based biomarkers of depression (Kambeitz et al. 2017). It is noteworthy that resting state functional connectivity has been proved to be superior classification accuracy of diagnostic models, compared with structural MRI or task-based fMRI data (Kambeitz et al. 2017).

Several studies have demonstrated the clinical value of resting state functional connectivity to distinguish MDD from healthy controls based on MVPA methods (Zeng et al. 2012; Zhong et al. 2017). The majority of the most discriminating functional connections were located within or across different resting state networks, such as DMN, SN and CEN, which were related to emotional and cognitive function (Zeng et al. 2012; Ma et al. 2013). In addition to methodological difference among these studies, the variable diagnostic performance may due to demographic and clinical characteristics of depressed patients. It was reported that antidepressant medicine and old age could cause alterations in brain function and structures (Anand et al. 2005; Guo et al. 2014c). Therefore, it was necessary and crucial to explore neuroimaging-based diagnostic models in first episode treatment-naïve young major depression. Though a few pioneering studies have now emerged on these topics, no final conclusion has yet been reached (Guo et al. 2014a; Zheng et al. 2019).

The aim of this study was to explore diagnostic models at an individual level to differentiate patients with MDD from healthy controls. MVPA and resting state functional connectivity were used as a diagnostic tool in first-episode, treatment-naive young adults with MDD and carefully matched healthy control subjects. We hypothesized that relative to healthy controls, abnormal cross-network functional connectivity were expected to be observed in resting state networks involved in emotional and cognitive function in MDD group.

## Materials and methods

### Participants

Patients with MDD were recruited from the psychiatric clinic at Xiangya Hospital of Central South University in Changsha, China. Patients with MDD were diagnosed according to the Structured Clinical Interview for DSM-IV by independent assessments of two psychiatrists. All of the patients were experiencing their first episode of depression and had never received medication. Closely matched healthy subjects were recruited through advertisements from several colleges in Changsha. All subjects were right-handed. In order to reduce the influence of addictive substance, all subjects were required to be abstinent from caffeine, nicotine, alcohol and other addictive substance at least one week prior to the fMRI scanning. The shared exclusion criteria for patients and control subjects included any major medical illnesses; clinical diagnosis of neurologic trauma; any history of psychiatric disorder in the control subjects or any history of psychiatric disorder, except major depression, in the MDD patients; any history of substance abuse or alcohol in the past 6 months; and any contraindications to imaging scanning. Finally, 30 patients with MDD and 31 matched healthy controls were recruited (Table 1).

**Table 1:** Demographic and clinical characteristics of the MDD and control groups

Characteristic	MDD	Control	t /χ^2^	p	Cohen’s d
Age	22.29 ± 1 .47	21.18 ± 3.32	0.43	0.72	0.15
Sex (female/male)	16/14	16/15	0.01	0.84	
CES-D	39.25 ± 5.75	18.33 ± 5.14	13.10	0	3.12
Age at onset (years)	21.17 ± 3.22	NA			
Illness duration (months)	8.35 ± 4.46	NA			

Written informed consent was obtained from all participants prior to the study, which was approved by the Institutional Review Board of Xiangya Hospital of Central South University for Brain Research. The methods were conducted in accordance with relevant approved guidelines and regulations.

### Measures

Depressive severity was measured using the CES-D scale (Radloff 1977), a 20-item self-report instrument to assess depressive symptoms in the general population. The Chinese version of the CES-D has been found to have high degrees of reliability and validity (Wang et al. 2013). In this study, the internal consistency of the CES-D was good (Cronbach’s alpha = 0.93).

### MRI data acquisition

Resting state fMRI images were captured by a 3T Siemens Magnetom Symphony scanner. During scanning, all participants were asked to rest with their eyes closed and to try not to think of anything systematically. All subjects placed their heads in a standard head coil (16-channel). Participants were positioned comfortably on the scanner bed and fitted with soft ear plugs; foam pad was used to minimize head movement.

Functional images were obtained axially using a single-shot, gradient-recalled echo-planar imaging sequence parallel to the line of the anterior-posterior commissure: repetition time/echo time = 2000/40 ms, thickness/gap = 5/0 mm, field of view = 240 × 240 mm, flip angle = 90°, matrix = 64 × 64, slices = 26, 150 volumes.

High-resolution T1-weighted images were also acquired with a three-dimensional spoiled gradient-recalled sequence in an axial orientation: repetition time = 8.5 msec, echo time = 3.2 ms, flip angle = 15°, field of view = 25 cm, matrix = 256 × 256, slice thickness = 1.0 mm, 176 slices.

### Image preprocessing

Image preprocessing was carried out using the Data Processing & Analysis for Brain Imaging software package (DPABI, http://rfmri.org/dpabi). After discarding of the first 10 volumes of each functional time series, slice timing, and realignment of head motion, data from three patients and two healthy subjects were excluded because their translation or rotation exceeded ±1.5 mm or ± 1.5°. The images were then spatially normalized to a standard template (Montreal Neurological Institute, Montreal, Quebec, Canada). The sources of spurious variance were regressed out including 6 parameters from head-motion correction (Friston 24-parameter model), white matter and cerebrospinal fluid signal. The resulting images were spatially smoothed with a Gaussian filter of 8 mm full-width half-maximum kernel. Linear detrending and temporal bandpass (0.01–0.08 Hz) filtering were performed to remove low-frequency drifts and physiological high-frequency noise. In view of the influence of head motion on functional connectivity results, the data was further performed with the scrubbing method to remove time points affected by head motions (Yan et al. 2013; Power et al. 2014).

### Anatomical parcellation

The registered functional MRI volumes with the Montreal Neurological Institute template were divided into 116 regions according to the automated anatomical labelling atlas (Tzourio-Mazoyer et al. 2002). The atlas divides the cerebrum into 90 regions (45 in each hemisphere) and divides the cerebellum into 26 regions (9 in each cerebellar hemisphere and 8 in the vermis). We evaluated functional connectivity between pairs of regions by calculating Pearson correlation coefficients. For each subject, we obtained a resting-state functional network captured by a 116 × 116 symmetric matrix. According to previous studies (Menon 2011, 2018; Supekar et al. 2019), these regions were divided into six different resting-state networks, such as DMN, SN, CEN, VN, Cerebellum and Other. The VN mainly comprised lingual gyrus, fusiform, cuneus and occipital lobe (Zeng et al. 2012).

### MVPA

#### Feature selection

Feature selection was used to construct the feature space for classification by retaining the most discriminating functional connections. The discriminative power of a feature can be quantitatively measured. The F score method was used for feature ranking in this current study for its simplity and effectiveness (Chen and Lin 2006), which has been widely used in mental disease (Liu et al. 2015; Chen et al. 2016) and other fields (Wang 2007; Akay 2009). *F* score of the *i*th feature is defined as follows:

$$ F(i)=\frac{{\left({\overline{x}}_i^{\left(+\right)}-{\overline{x}}_i\right)}^2+{\left({\overline{x}}_i^{\left(-\right)}-{\overline{x}}_i\right)}^2}{\frac{1}{n_{+}-1}{\sum}_{k=1}^{n_{+}}{\left({\overline{x}}_{k,i}^{\left(+\right)}-{\overline{x}}_i^{\left(+\right)}\right)}^2+\frac{1}{n_{-}-1}{\sum}_{k=1}^{n_{-}}{\left({\overline{x}}_{k,i}^{\left(-\right)}-{\overline{x}}_i^{\left(-\right)}\right)}^2} $$

*where*${\overline{\ x}}_i$ is the average of the *i*th feature of the whole data set*,* ${\overline{x}}_i^{\left(+\right)}\ \mathrm{and}\kern0.5em {\overline{x}}_i^{\left(-\right)}$are the averages of the *i*th feature of MDD and healthy control data sets;${x}_{k,i}^{\left(+\right)}$is the *i*th feature of the *k*th MDD instance, and ${x}_{k,i}^{\left(-\right)}$is the *i*th feature of the *k*th healthy control instance*.* The denominator represents the discrimination within each of the MDD and healthy control sets, while the numerator represents the discrimination between the two sets. Obviously, the larger the *F* score is, the more likely the feature is of more potential to discriminate the groups.

### Support vector machine classification

Support vector machine (SVM) classifier was adopted for classification, which works well when the number of training samples is small but the number of features is large (Vapnik 2000). SVM classification is one type of supervised learning which consists of two steps: training and testing. During the training step, SVM forms the decision function from the training data set with its class labels. During the testing step, it predicts the class labels of new test examples (Liu et al. 2015). A linear kernel SVM was used, in order to reduce the risk of overfitting the data and allow direct extraction of the feature weights (Pereira et al. 2009). The SVM classifier was implemented using LIBSVM toolbox with default parameters (Chang and Lin 2011).

### Evaluation of the performance of the classifier

Due to our limited number of samples, a LOOCV strategy was employed to evaluate the performance of the classifier (Scholkopf and Smola 2001; Liu et al. 2015; Chen et al. 2016). In brief, suppose there were *n* samples in total. In each LOOCV trial, *n*-1 samples were used as the training set and the remaining one was used as the testing set. This procedure was repeated *n* trials. Classifiers were built for each training set and tested with its corresponding testing subject. Accuracy, sensitivity, and specificity could be used to quantify the performance of the classifier based on the results of LOOCV. Utilizing of LOOCV strategy could get stable weights of each feature and the weights got from the training dataset were more close to the whole dataset (Anderson et al. 2014).

### Classification weight definition

In each trial of LOOCV, the final features used in classification differed because feature ranking was based on a slightly different subset of the data. Consensus features were defined (Liu et al. 2015; Chen et al. 2016). They were regarded as the common features always selected to form the final features set from each LOOCV iteration. The weight of the consensus feature was the average value of the classification weight across all trials of LOOCV. The weight of a consensus feature was defined as zero if this connection was not selected as a classification feature.

To represent the relative contribution of different regions for classification, the classification weight of each region was evaluated by summing one-half of the classification weight of the connections associated with that region (splitting the weight of connections into the regions they connects) (Meier et al. 2012). Of note, if a region did not form any consensus feature, it was given a region weight of zero. We defined a region (consensus feature) with greater weights if its weight was at least 1 standard deviation greater than the average of the weight of all the regions (consensus features) (Tian et al. 2011; Liu et al. 2015).

### Permutation test of classification performance

To estimate the statistical significance of observed classification accuracy is a challenging problem due to the high dimensionality of the fMRI data and the relatively small number of training examples. Some researchers have proposed a framework of permutation test, which is a nonparametric technique in which a reference distribution is obtained by calculating all possible values of the test statistic under rearrangements of the labels of the samples (Golland and Fischl 2003). The permutation test has widely used in classifying brain states (Mourao-Miranda et al. 2005), sexual dimorphism (Wang et al. 2012) and resting-state brain function (Zhu et al. 2008; Liu et al. 2015). In the current analysis, the class labels of the training data were randomly permuted 1000 times. The same entire classification process including feature selection was carried out with each set of permuted class labels. The accuracies were obtained across all permutations. Based on these null probability distributions and the observed statistic corresponding to the actual labeling, *p* value was calculated as the proportion of accuracies that are equal to or greater than the accuracy obtained by the non-permutated (original) data (Liu et al. 2015). The smaller is the *p* value, the more reasonable to reject the null hypothesis. Usually a threshold of *p <* 0.05 is meaningful.

## Result

**Fig. 1:**
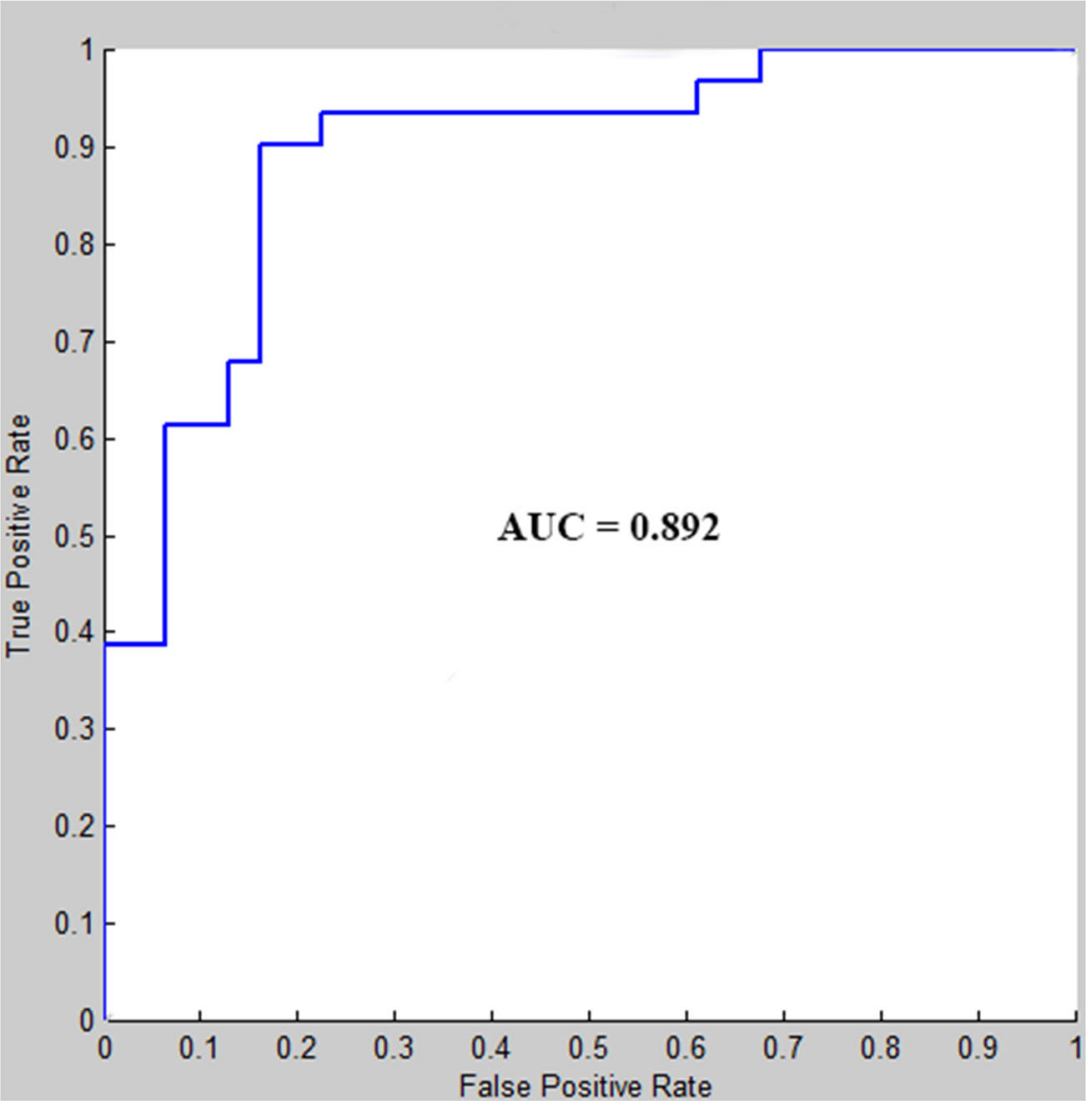
ROC curve of the classifier

A relatively high classification accuracy of 82.25% was achieved in this study (sensitivity 83.87%, specificity 80.64%, *p* < 0.001). The receiver operating characteristic (ROC) curve of the classifier was shown in Fig. 1. The area under the ROC curve (AUC) was 0.892, indicating a good classification power.

**Fig. 2:**
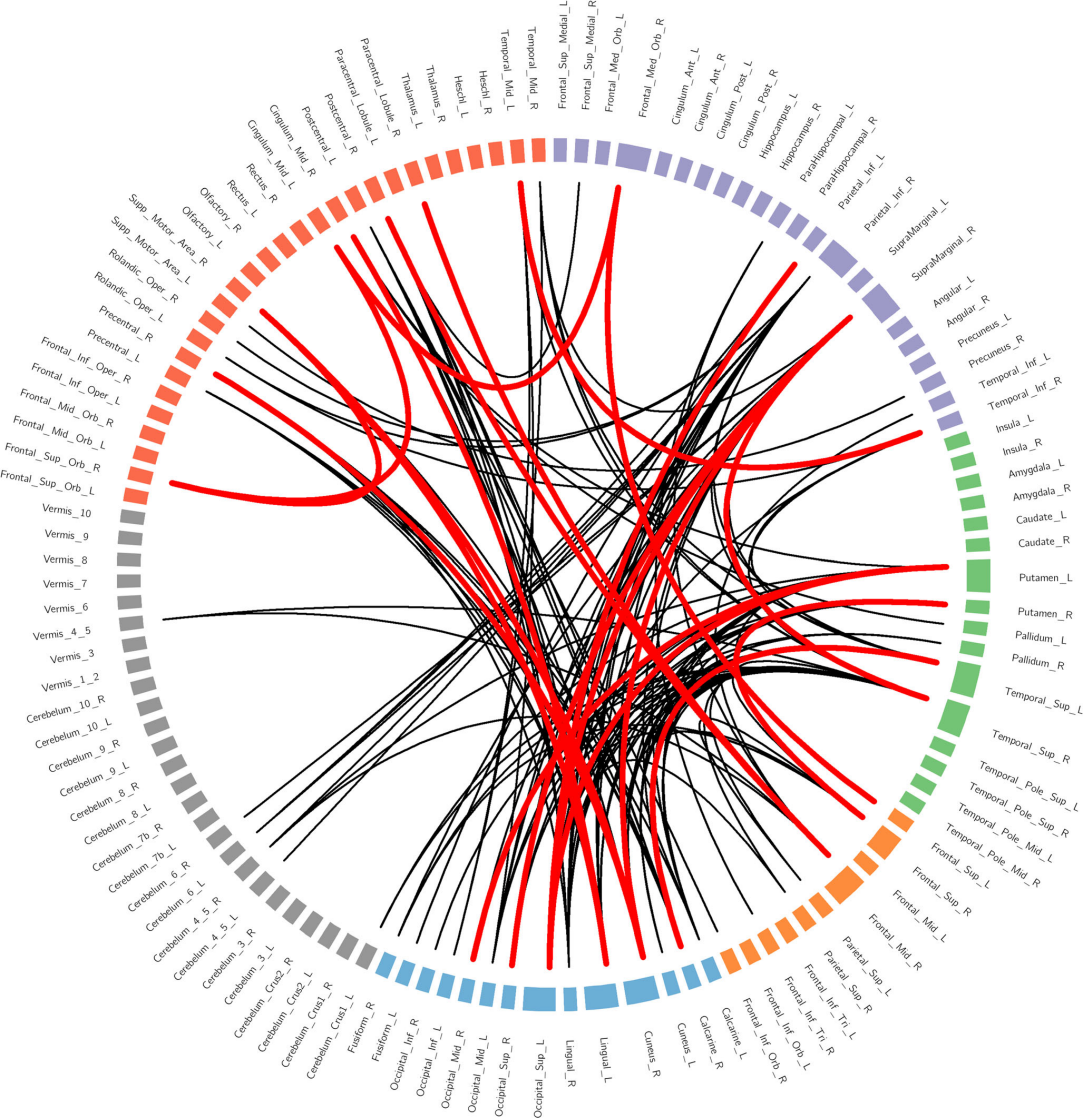
Regions and distribution of 128 consensus features. Different colors of nodes belong to different networks. Purple means default mode network (DMN), green means salience network (SN), orange means central executive network (CEN), blue means visual cortical network (VN), grey means Cerebellum network, red means Other network. Red lines represent the connections with more classification weight, and black lines represent the connections whose classification weight were under the mean plus/minus the standard error of all connections used as classification features

128 consensus features were identified in the cross-validation. Similar to the previous studies, brain regions related to consensus functional connectivity were found to be located primarily in 6 resting state networks: DMN, SN, CEN, VN, Cerebellum and Other. Almost the entire consensus connections (125/128) used to distinguish MDD from healthy controls belonged to the cross-network interaction. Some consensus features exhibited greater weights than others, which means that its weight was at least one standard deviation greater than average weight of all the regions (Tian et al. 2011). 128 consensus features were shown in Fig. 2.

**Fig. 3:**
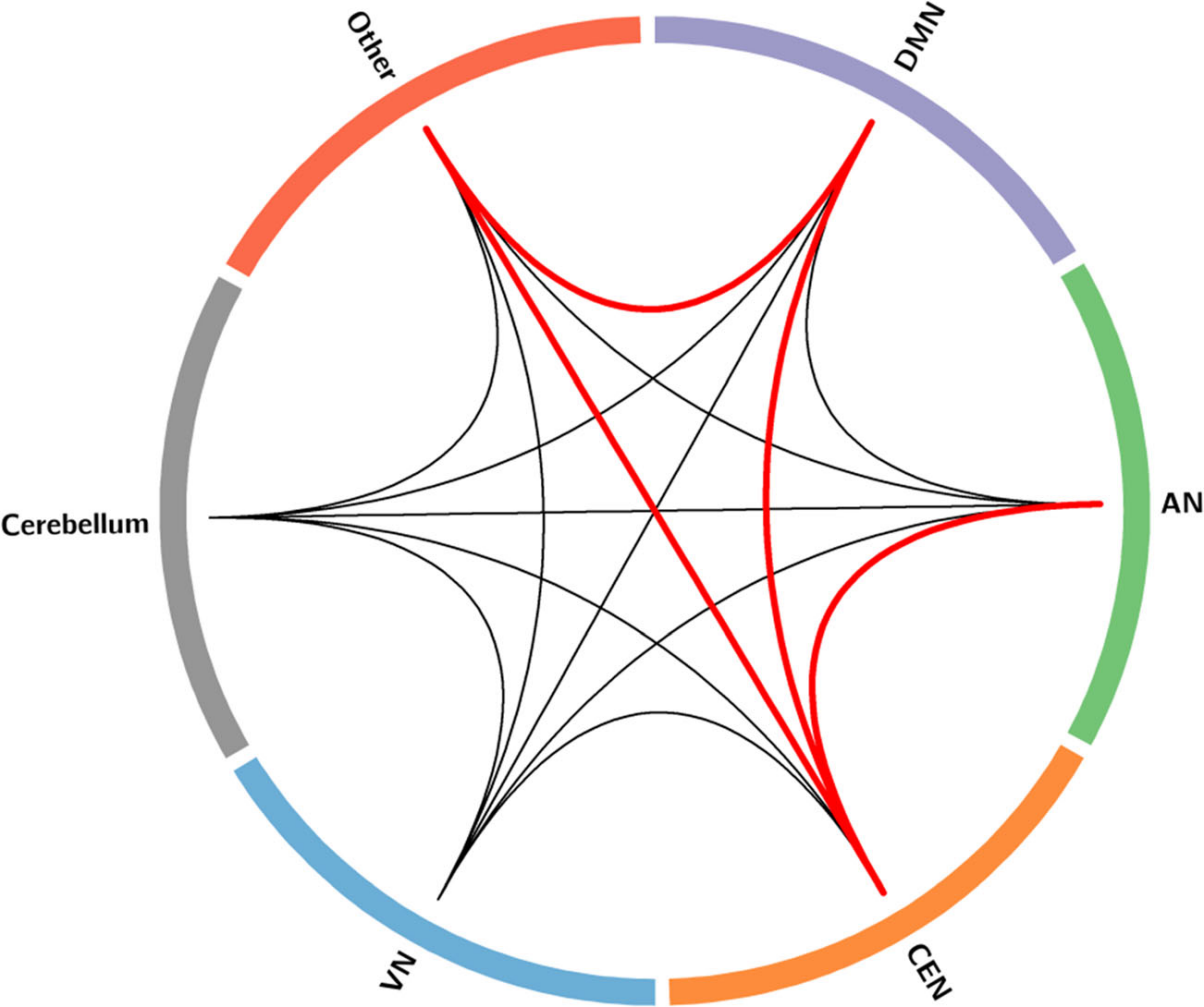
Cross-network interaction among 6 resting state networks. Red lines represent connections with greater weight than mean weight plus the standard error of all connections; black lines represent left connection except the greater connections

The mean weight of consensus features across two networks is one indicator to represent the role of interactions across these two networks to some extent. Therefore, the mean weight of consensus features cross 6 networks was calculated. Cross-network interactions in 6 networks were constructed in Fig. 3. Some cross-network interactions exhibited greater weights than others. The cross-network interactions with greater weight were mainly located across DMN, SN, CEN and VN.

**Fig. 4:**
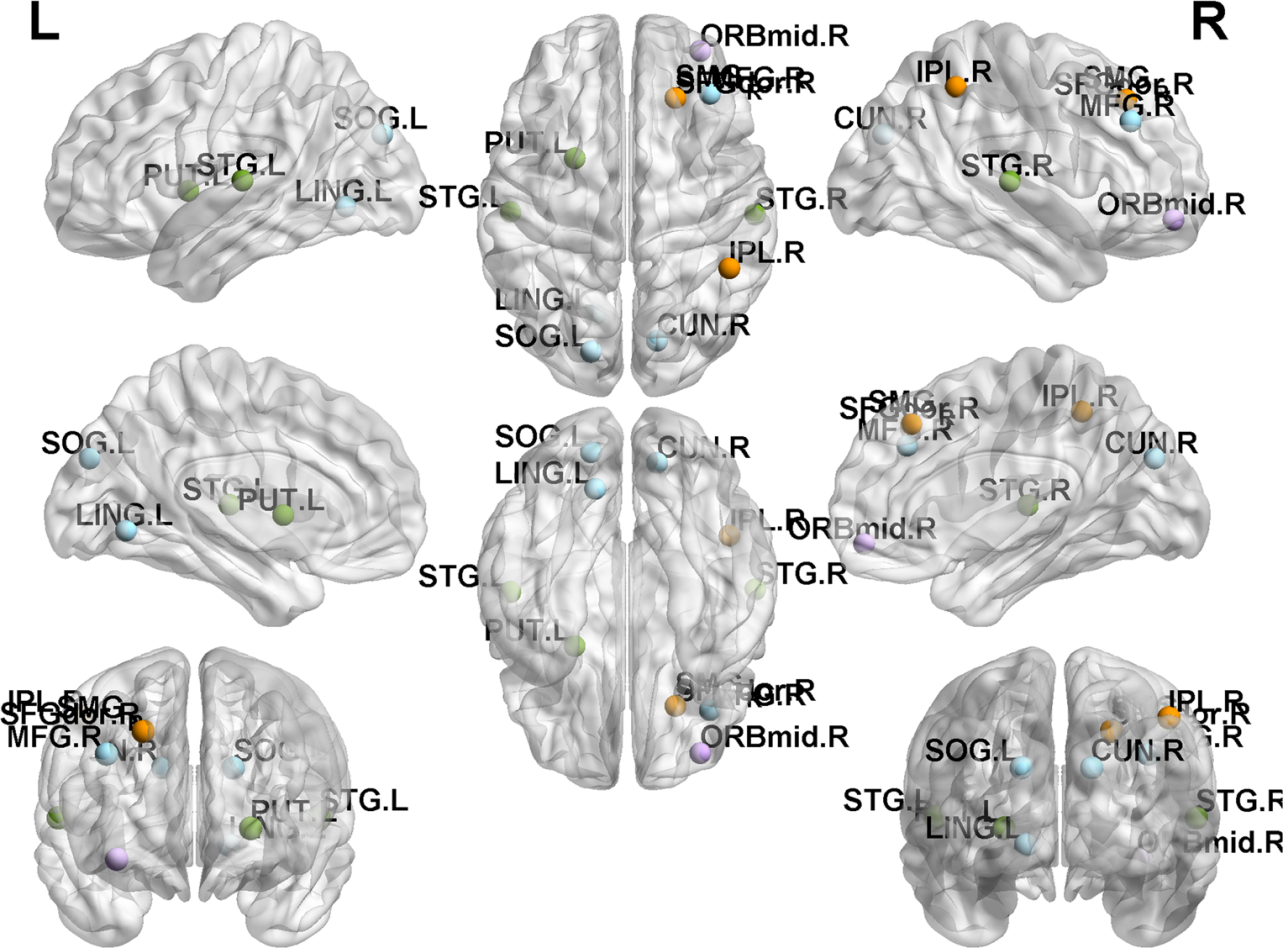
Nodes with greater classification weight which is higher the mean plus the standard error of all connections. Nodes with purple color were located in DMN, green in SN, orange in CEN, blue in VN, grey in Cerebellum, and red means Other network

Several brain regions exhibited greater weights than others. These regions contain right supramarginal gyrus and right infer parietal lobule (involved in DMN), super temporal gyrus and left putamen (involved in SN), left super occipital gyrus, and lingual gyrus (involved in VN). Of all, the supramarginal gyrus exhibited highest discriminative power. Figure 4 showed these regions.

## Discussion

In this study, resting state functional connectivity was used as the feature to identify first-episode, drug-naïve MDD patients from health populations using MVPA methods. Four main results were revealed: (1) a correct classification rate was 82.25% and the AUC value was 0.892, indicating the important value of whole brain resting state functional connectivity to identify MDD patients from healthy controls; (2) almost all of the consensus connections (125/128) used to distinguish MDD belonged to cross-network connection among DMN, SN, CEN, VN, Cerebellum and Other; (3) The consensus connections with greater weight were mainly located across DMN, SN, CEN and VN. (4) The supramarginal gyrus exhibited the highest discriminative power.

Consistent with previous findings, cross-network interaction was found to be altered in patients with MDD. DMN, SN, CEN, VN and Cerebellum have been commonly regarded as key resting state networks in MDD, with abnormalities having been observed in blood oxygenation level-dependent fMRI activation, as well as in baseline metabolism or perfusion (Gong and He 2015; Mulders et al. 2015). However, it should be noted that the networks did not function independently. It may be not enough to only investigate the connectivity within one specific network. In addition to the within-network connectivity, cross-network connectivity was also investigated by a large number of studies on resting-state functional connectivity in MDD (Brakowski et al. 2017). The interplay between the DMN and CEN and their sub-networks have been hot topics in this field (Mantini et al. 2007; Manoliu et al. 2014b; Zhu et al. 2017; Liu et al. 2018). A meta-analysis study suggested that altered connectivity between neural systems involved in cognitive control and those that support salience or emotion processing may relate to deficits regulating mood in MDD (Kaiser et al. 2015). Our study extends these prior findings by providing new evidence for abnormal resting-state functional connectivity in MDD.

It is worth noting that aberrant functional organization of DMN, SN and CEN was observed in the resting-state data of MDD subjects. The recently proposed “triple-network” model emphasized the corporation among these three networks, which has been revealed to underlie a wide range of psychopathologies, including schizophrenia, autism and attention-deficit/hyperactivity disorder (Bressler and Menon 2010; Menon 2011). Dysfunction of three networks has remarkably occurred in many mental and neurological disorders (Manoliu et al. 2014b). With a similar pathophysiological mechanism observed in schizophrenia, MDD is reported to demonstrate common causal dysconnectivity between DMN and SN, as well as opposing functional dysconnectivity of DMN-CEN and SN-CEN (Jiang et al. 2017). Significantly decreased interaction degree between DMN and CEN was reported in MDD (Zheng et al. 2015). Consistent with previous studies, our study suggested that the abnormal triple networks interaction in resting state of MDD patients.

As a part of DMN, the supramarginal gyrus showed highest classification weight in present study. Great difference in the functional connectivity of the DMN between individuals with MDD and healthy controls has been revealed (Greicius et al. 2007; Zhu et al. 2012; Hamilton et al. 2015). As anterior to the junction of parietal and temporal cortex, the supramarginal gyrus is traditionally known to be involved in several cognitive functions, including speech repetition, auditory short-term memory (Buchsbaum and D'Esposito 2009; Baldo et al. 2012). However, recent converging evidence from multiple methods and experiments that the supramarginal gyrus is crucial for overcoming emotional egocentricity in social judgement, which is closely associated with self-referential processing (Silani et al. 2013). Damage of supramarginal gyrus and adjacent areas can produce a variety of disorders associated with distorted body knowledge and self-awareness (Berlucchi and Aglioti 1997).

Although the classification accuracy of this present study was favorable, several limitations should be noted. As well as many previous studies in this field (Zeng et al. 2012; Liu et al. 2015; Zhong et al. 2017), the first limitation is related to small sample size with no comorbid conditions, so we may be cautious in generalizing the findings of this study to more larger samples with comorbid diagnoses. In the future, larger sample size, multicenter imaging data and a large independent test data set are welcome to confirm the classification results. Secondly, we only explored resting state functional connectivity and did not consider brain structural connectivity, dynamic functional connectivity (Li et al. 2019; Liao et al. 2019) or combined dynamic and static connectivity (Liao et al. 2018). Functional and structural, static or dynamic imaging data will be combined to provide more reliable diagnostic information. Thirdly, automated anatomical labeling atlas was used in this study. Previous studies revealed that different templates could impact the generated connections at a certain degree, more brain templates would be used to confirm the accuracy.

## Conclusion

In summary, we have demonstrated multivariate pattern analysis methods can identify first episode drug-naive patients with MDD from healthy controls based on resting-state functional connectivity with a correct classification rate of 82.25% (*p* < 0.001, sensitivity 83.87%, specificity 80.64%). Almost all of the most discriminating consensus connections were cross-network connectivity among DMN, SN, CEN, VN, Cerebellum and Other network, which implied the emotional and cognitive impairments characteristic of MDD. Moreover, the supramarginal gyrus located in DMN exhibited the highest discriminative power in classification. The current study further confirmed reliable application of MVPA in the discriminating MDD patients from healthy controls. More importantly, these results support the cross-network interaction as an effective biomarker for MDD clinical diagnosis, which may reveal the potential pathological mechanism for major depression.
